# The effect of lutein and *Urtica dioica* extract on in vitro production of embryo and oxidative status in polycystic ovary syndrome in a model of mice

**DOI:** 10.1186/s12906-021-03229-x

**Published:** 2021-02-08

**Authors:** E. Bandariyan, A. Mogheiseh, A. Ahmadi

**Affiliations:** 1 Department of Clinical Sciences, School of Veterinary Medicine, Shiraz University, P.O. Box. 7144169155, Shiraz, Fars Iran; 2 Department of Basic Sciences, School of Veterinary Medicine, Urmia University, Urmia, West Azerbaijan Iran

**Keywords:** Lutein, Nettle extract, Antioxidant, Mice, Embryo

## Abstract

**Background:**

Polycystic ovary syndrome (PCOS) is one of the most prevalent endocrinopathies in women during the reproductive age. Herbal medicines are used increasingly alone or in supplement with chemical medicines for the treatment of different diseases and dysfunctions. This study was aimed to evaluate the effects of lutein and nettle (*Urtica dioica*) extract on the biochemical parameters and the reproductive function in the PCOS model of mice.

**Methods:**

Following the induction of PCOS by dehydroepiandrosterone (DHEA), the mice (*n* = 98) were randomly assigned into seven groups, each consisting of fourteen mice; the groups were included control group (received solvent), PCOS group (received 6 mg/100 g B.W/day IP, DHEA for 21 days), PCOS+ Nettle extract (200 and 400 mg/kg), PCOS+ Lutein (125 and 250 mg/kg), and PCOS+ NL (200 mg/kg nettle extract and 125 mg/kg lutein). The nettle extract and lutein were administrated using gavage for 30 consecutive days after PCOS induction. Malondialdehyde (MDA), total antioxidant capacity (TAC), and estrogen were measured in serum, ovary, and uterus samples by the ELISA method. The total number of oocytes, oocyte quality, fertilization rate, 2-cell blastocyst, and arrested embryos (type I, type II, and type III) were also investigated.

**Results:**

A combination treatment of the nettle and lutein produced the lowest concentration of MDA in comparison to other groups which affected by the PCOS. The lowest level of TAC was observed in the PCOS group without treatment. The number of oocytes, oocyte quality, fertilization rate, and 2-cell blastocyst were significantly higher in the control group, but the lowest values were observed in the PCOS group without any treatment.

**Conclusions:**

The most favorable findings include improving antioxidant capacity, oocyte and embryo quality were observed in the PCOS+ 125 L group.

## Background

Polycystic ovary syndrome (PCOS) has been reported as one of the most prevalent endocrinopathies in women during the reproductive age [1]. PCOS is known as one of the metabolic disorders with reproductive side effects [2]. The PCOS is a complex disorder due to the high amount of androgens, irregularities in the endometrium, and also some cysts on ovaries. PCOS has been known to have a relationship with metabolic disorders, such as obesity, insulin resistance [3], hyperinsulinemia, and type 2 diabetes mellitus [4]. PCOS has been associated with three phenotype properties including hyperandrogenism, polycystic ovaries, and ovulatory dysfunction [5]. The ovulatory dysfunction often requires pharmacological intervention for improving ovulation and conception [6]. Aromatase has been known as a granulosa cell enzyme that converts androgens to estrogens. It has been accepted that there is decreased aromatase activity in women with PCOS [7]. Hyperandrogenism could be attributed to decreased aromatase activity [8, 9]. Increased androgens could affect follicular development negatively and prevent meiotic maturation by reducing the fluctuations of intracytoplasmic calcium levels [10]. On the other hand, oxidative stress has been reported to be associated with PCOS [11]. The circulating markers of oxidative stress were abnormal in women with PCOS. It means that oxidative stress could have a role in the pathophysiology of PCOS [12]. Several factors such as formation of reactive oxygen species (ROS) are involved in low-quality of embryos following in vitro culture. ROS blocks meiosis in oocytes and prevents embryonic development and induces cell death. Different antioxidants are applied to mitigate the negative effects of ROS in embryos [13]. The formation of free radicals exceeds the embryos’ antioxidant capacity during in vitro culture of embryo in mammals; accordingly, different exogenous antioxidants are used to overcome the imbalance oxidative conditions [14].

Herbal medicines have been used for the treatment of different diseases and dysfunctions since a long time ago. The World Health Organization recommends the use of medicinal plants and encourages researchers to define the rational use of medicinal plants as a source of novel therapeutic agents. The medicinal plants have been appropriate options for the treatment of infertile couples [15]. Nowadays, using herbal treatment is increasing due to the widespread drug resistance and the side effects and high costs of chemical drugs [16].

Lutein is a dietary carotenoid which is extracted from dark green leafy vegetables, oranges, yellow fruits, and vegetables [17]. Lutein cannot be synthesized in mammals and it must be supplied in the diet for use by different tissues [18]. In vivo (mouse, rat) and in vitro (cell cultures) studies indicated anti-inflammatory, antioxidant, anti-apoptotic and modulating lipid metabolism effects of lutein in the eye, ear, coronary artery, heart and spinal cord. Its effects observed in serum, tissue and gene levels were related to lipid metabolism, inflammation, oxidative stress and apoptosis [19–27]. The ovary is metabolically a very active organ and produces a high level of oxidants. Oxidative stress and inflammation play critical roles in pathophysiology of PCOS [28, 29]. Lutein may decrease the formation of cystic follicles on the ovary by its antioxidant and anti-inflammatory abilities.

*Urtica dioica* belongs to the Urticaceae family and is referred to as stinging nettle. Nettle has been classified under the group of key plants in the European pharmacopoeia. It is known to have some pharmacological properties, including antioxidant [30], anti-inflammatory, antiulcer [31], anticancer [32], antibacterial, and antifungal [33] properties. Rat, mice, dog, chicken as animal models and cell culture have been used for evaluating the effects of nettle extract on controlling inflammatory cytokines and clinical signs, immunological response, blood glucose and glucose transporter gene and lipid peroxidation in different organs [34–38]. Nettle extract was effective in controlling morphological and histological changes in polycystic ovaries and complications of metabolic syndrome modification of sex hormones in rat model of PCOS [39].

Both lutein and nettle have antioxidant and anti-inflammatory properties, which could be helpful in the PCOS condition. Therefore, this study was conducted to evaluate the effects of lutein and nettle extract on biochemical parameters and in vitro production of embryos in the PCOS model of mice. We expected that the use of lutein and nettle extract could mitigate the negative effects of PCOS on the fertility of mice suffering from this syndrome.

## Methods

Experimental protocols were performed in accordance with the Iranian animal ethics framework and under the supervision of the Iranian Society for the Prevention of Cruelty to Animals and Shiraz University Research Council (IACUC no: 4687/63).

### Preparation of nettle leaves extract

During April 2018, the aerial parts of nettle were obtained from wild grasslands around Shiraz, Fars Province. Afterward, the achieved aerial parts were washed and dried in 55 °C using an oven. The dried parts were then powdered and mixed with ethanol in a ratio of 1:10; the extraction was conducted in darkness. The extract was filtered by clean cotton. Following the preparation of hydroalcoholic extract, it was evaporated to remove the ethanol at less than 40 °C using a rotary evaporator and stored at − 20 °C until future use. The plant species was identified and authenticated by A. R. Khosravi, a plant taxonomist at Shiraz University Herbarium, Shiraz, Iran. Voucher specimen (PM 533) of this material has been deposited in a herbarium. Lutein was purchased from the Biochem Company (Irvine, CA 92618, U.S.A).

### Animals and experimental design

**Fig. 1:**
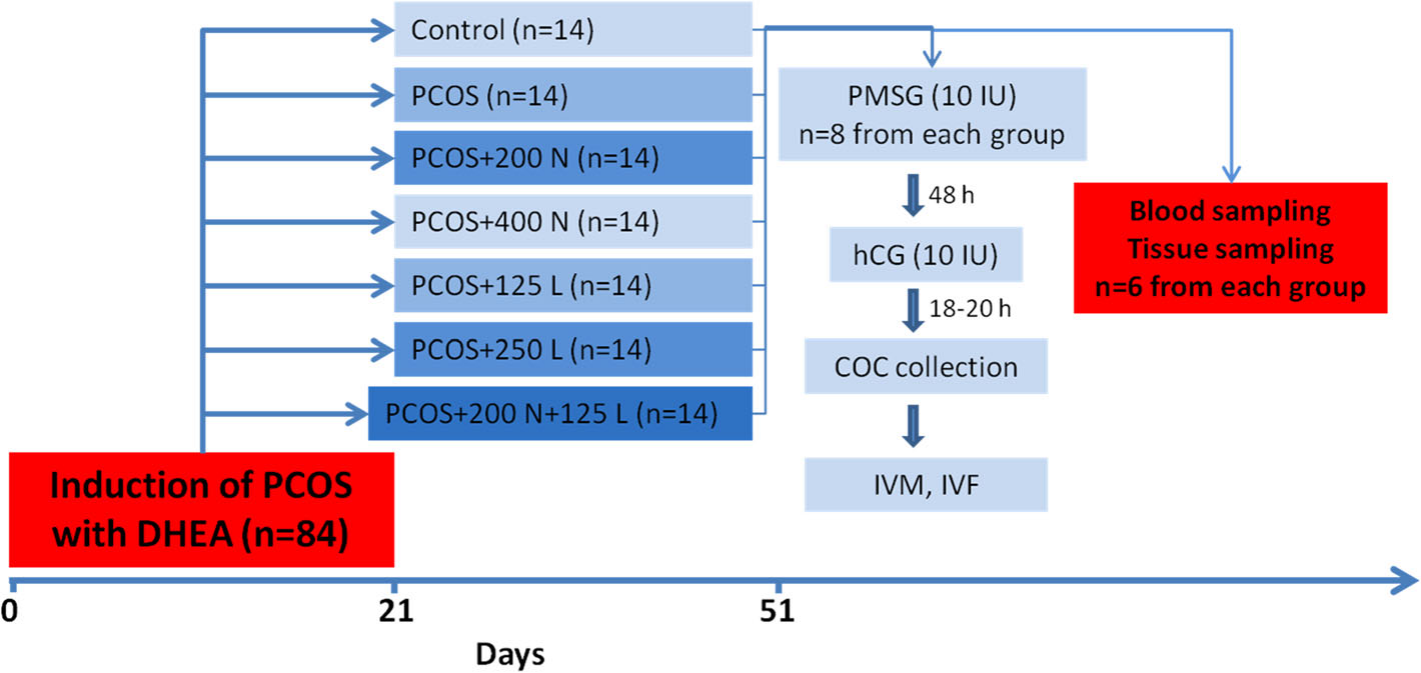
Schematic diagram of the study design. PCOS was induced in the first 30 days in 84 mice. Different treatments were administrated orally within the second 30 days. Eight mice from each group were aligned for superovulation, COCs collection, IVM, IVF, and IVC program. Blood and tissue samples were collected from six mice from each group. Polycystic ovary syndrome (PCOS); Dehydroepiandrosterone (DHEA); Lutein (L); Nettle extract (N)

A total of 98 NMRI mice, aging 20-day old and weighing 14–17 g, were used in this study. All the animals were purchased from the animal laboratory of Urmia Medical School, Urmia, Iran, and quarantined for 14 days. The mice were grouped in polycarbonate cages at 21–24 °C, 40–45% humidity, and 12 h light and dark cycles at Laboratory Animals Breeding in Urmia University. This study was performed in accordance with the directions and guidelines for the care and use of laboratory animals. To induce the PCOS, dehydroepiandrosterone (DHEA) (6 mg/100 g B.W/day; Sigma-Aldrich Co.) was administered intraperitoneally for 21 consecutive days [40] in all the groups (*n* = 84), except that for the control group (*n* = 14). The mice were randomly assigned into seven groups, each consisting of fourteen mice. The mice were assigned in a group in which PCOS was not induced (Control), a group with induced PCOS (PCOS), the PCOS mice group which was administrated 200 mg/kg nettle extract (PCOS+ 200 N) [41], the PCOS mice group which was administrated 400 mg/kg nettle extract (PCOS+ 400 N) [42], the PCOS mice group which was administrated 125 mg/kg, lutein (PCOS+ 125 L) [41], the PCOS mice group which was administrated 250 mg/kg, lutein (PCOS+ 250 L) [41], and the PCOS mice group which was administrated 200 mg/kg nettle extract and 125 mg/kg lutein (PCOS+NL). The mice were received lutein and nettle extract in gavage form daily, for the next 30 days after induction of PCOS period [43] (Fig. 1). The induced PCOS syndrome was confirmed by weight gain, vaginal smear (at days 21 and 51), and polycystic ovary appearance at the end of the treatment period (day 51).

### Superovulation and in vitro fertilization (IVF)

**Fig. 2:**
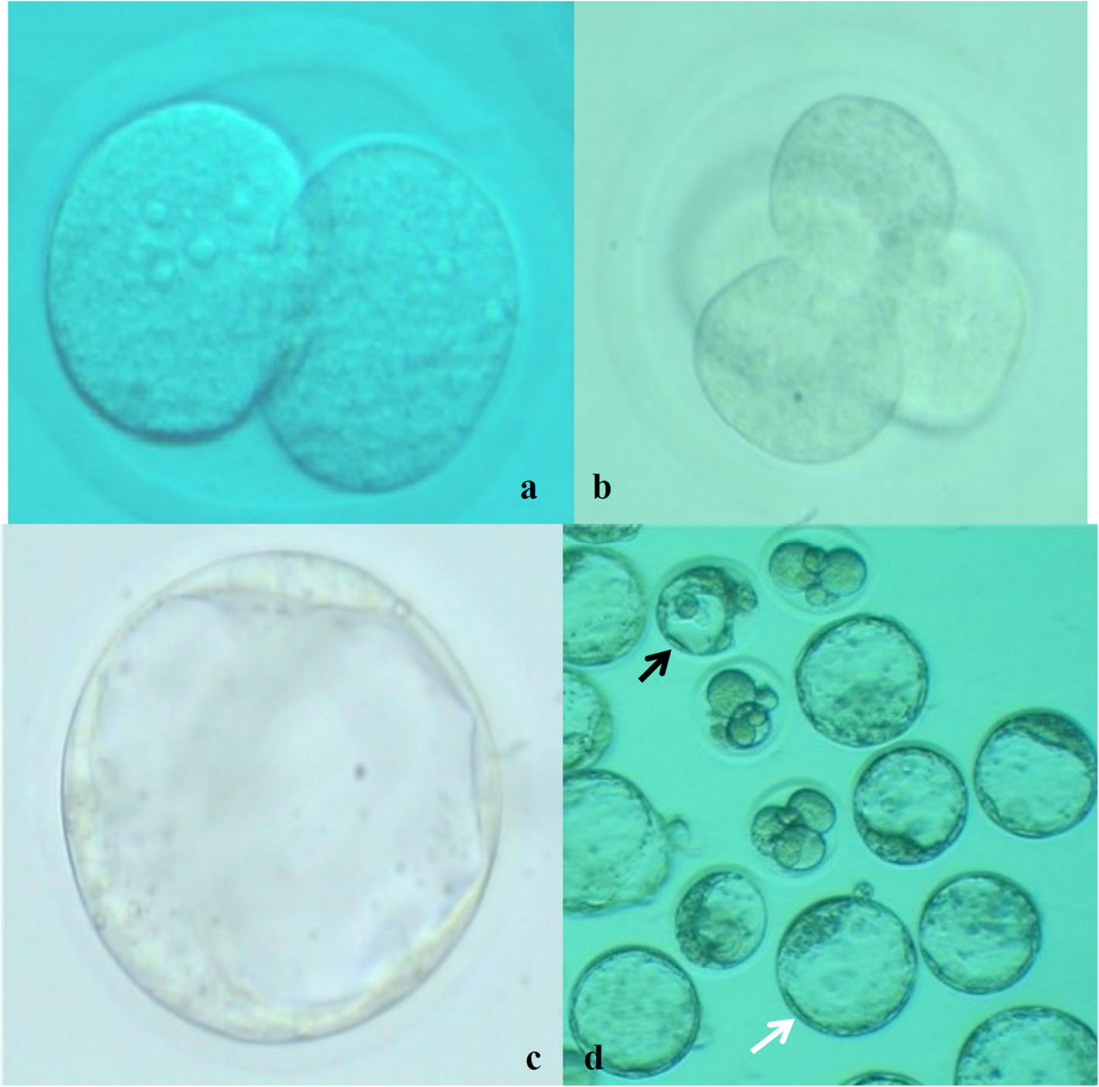
The mouse embryos obtained during 120 h of in vitro culture in CO_2_ incubator. **a** Two-cell embryo (× 200); **b** four-cell embryo (× 200); **c** blastocyst embryo (× 200); **d** normal and abnormal blastocyst embryos (× 100), black arrow: arrested embryo, white arrow: normal blastocyst

After the treatment period, on day 51, to stimulate the superovulation, 10 IU of pregnant mare serum gonadotropin (PMSG, Folligon®, Intervet, France) was injected intraperitoneally, followed by the intraperitoneal injection of 10 IU Human Chorionic Gonadotropin (hCG, Folignan, Daropakhsh, Iran) after 48 h in eight mice from each group. The mice were sacrificed 13 h after the injection of hCG; then, their oviduct was removed (Fig. 1). The MII oocytes were removed when the ampule of the oviduct was ruptured. The MII oocytes were washed in one droplet and transferred to another plate, containing the droplets for IVF. After extraction and washing of the oviducts, the cumulus-oocyte complexes were transferred to fertilization medium droplets under mineral oil containing HTF culture medium with 4 mg / ml BSA (Sigma, St. Louis, USA). The epididymal sperm was collected from the caudal epididymis of a male adult mouse. Sperm suspensions were positioned in HTF+ 4 mg/ml BSA medium and capacitated through incubation at 37 °C and 5% CO2 for at least 60 min [44]. Then, 1 × 10^6^ sperm/ml was added into 500 μL fertilization droplets of the HTF-BSA medium having oocytes. Mineral oil was applied for covering the droplets. The mineral oil was washed by adding the same culture medium and then, in order to reach equilibrium, it was placed in an incubator with CO2 for 12 h. Under an inverted microscope, the fertilized oocytes were evaluated and confirmed by the presence of male and female pronuclei and second polar body. Following 120 h of zygotes culture, the total number of oocytes, oocyte quality (oocytes were classified based on their morphological characteristics including the polar body extrusion, cumulus cell layer, zona pellucida, and perivitelline space), fertilization rate, 2-cell blastocyst, and arrested embryos (type I, type II, and type III) were evaluated in each group by a reverse microscope. The scoring of the arrested embryos was performed on the basis of the rate of lysis and necrotic changes as follows: Type I: lysis-based embryos, fragmented and completely necrotic; Type II: embryos with lysis and fragmentation in some blastomeres; Type III: embryos with a small number of lysed and fragmented blastomeres and cytoplasmic vesicles [45] (Fig. 2).

### Biochemical analysis

Twenty-four hours after the last treatment, six of the mice from each group were intraperitoneally anesthetized with xylazine (10 mg/kg) and ketamine (50 mg/kg) [46]. Blood samples were obtained immediately after euthanasia with decapitation of deeply sedated mice, prepared in centrifuge tubes without anticoagulants, and allowed to clot. The blood samples were then centrifuged in 3000 × rpm for 20 min. Sera samples were separated and then quickly stored at − 80 °C for biochemical analyses. Malondialdehyde (MDA), total antioxidant capacity (TAC) (Zell Bio GmbH, Germany), and estradiol-17β hormone (Monobind Inc., Lake Forest, CA, USA) were measured using commercial ELISA kits according to the manufacturer’s protocols.

### Ovary and uterus investigations

Euthanasia was performed by decapitation of mice were deeply anesthetized with combination of xylazin and ketamini [47]. Uterus and ovary samples were separated from six mice from each group and investigated in terms of TAC and MDA (ZellBio GmbH, Germany) and estradiol-17β hormone (Monobind Inc., Lake Forest, CA, USA).

### Statistical analysis

The results of IVF were analyzed by 2 proportion tests (Chi-Squared) using Minitab software version 15.1 (Minitab Inc., PA, USA). The data was tested using the Shapiro–Wilk test for normality. Estradiol-17β, MDA and TAC results were compared among groups by one-way ANOVA and Tukey’s post-hoc tests using SPSS software (Statistical Package for the Social Sciences, version 16, SPSS Inc., Chicago, Illinois, USA). Figures were illustrated by Graph Pad Prism software. All results were shown as means ± standard deviation (SD), and a *P* < 0.05 was determined as statistically significant.

## Results

Vaginal cytology examination revealed the PCOS mice were interrupted in metestrus phase and also, the control group was in the metestrus stage in spite of regular estrous cycle (about every 5 days) at days 21 and 51.

### Estradiol-17β concentration in the serum, uterine, and ovary samples (Fig. 3)

Serum estradiol-17β concentration: Treatment with lutein and nettle extract could significantly decrease the levels of estradiol-17β in comparison to those of the PCOS groups (*P* < 0.0001). The control group showed the lowest levels of estradiol-17β (34.25 ± 1.70 pg/ml). In comparison to the higher doses of lutein (250 mg/kg) (43.50 ± 1.29 pg/ml) and nettle (400 mg/kg) (46.50 ± 1.27 pg/ml), the administration of lower doses of lutein (125 mg/kg) (51.25 ± 1.70 pg/ml) and nettle (200 mg/kg) (52.50 ± 1.29 pg/ml) produced higher levels of estradiol-17β. Combined treatment with lutein and nettle extract decreased estradiol-17β to the lowest concentration (37.50 ± 1.35 pg/ml) in comparison with that of other groups.

**Fig. 3:**
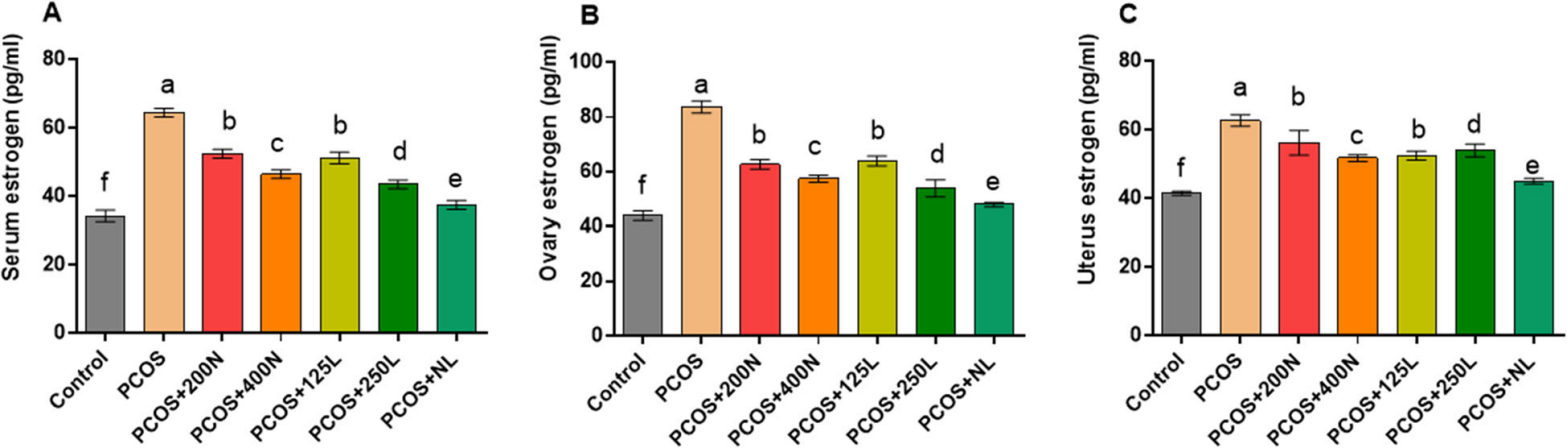
Estradiol-17β concentration in the serum, ovary, and uterine tissue samples are presented and compared in different groups of mice (*n* = 14)

Ovary estradiol-17β concentration: The lowest estradiol-17β levels were observed in the control group (44.00 ± 1.82 pg/ml) while the highest level was recorded in the PCOS group (83.75 ± 2.21 pg/ml). All treatment groups showed a significant difference when compared to the control and PCOS groups (*P* < 0.05). The most favorable treatment result (decreased estradiol-17β) was seen in the PCOS+NL group. Among the treatment groups, there was no significant difference between the PCOS+ N125 and PCOS+ N200 groups.

Uterus estradiol-17β concentration: The results showed that the lowest level (41.50 ± 0.577 pg/ml) of serum and ovary estradiol-17β levels belonged to the control group and the highest level was found in the PCOS group (62.75 ± 1.70 pg/ml). Despite the apparent difference between the PCOS+ 200 N and PCOS+ 125 L groups, the difference was not significant. Among treatment groups, the lowest level of estradiol-17β was observed in the PCOS+NL group, which had a significant difference with all other treatment groups (*P* < 0.05).

### MDA concentration in the serum, uterine, and ovary samples (Fig. 4)

Serum MDA level: The PCOS group showed the highest levels of MDA (3.07 ± 0.12 μmol/L) in comparison with that of other groups. Higher doses of lutein produced lower levels of MDA. Similar to the findings regarding estradiol 17β, a combination of the nettle and lutein led to the lowest values of MDA in comparison to those of other mice affected by PCOS.

**Fig. 4:**
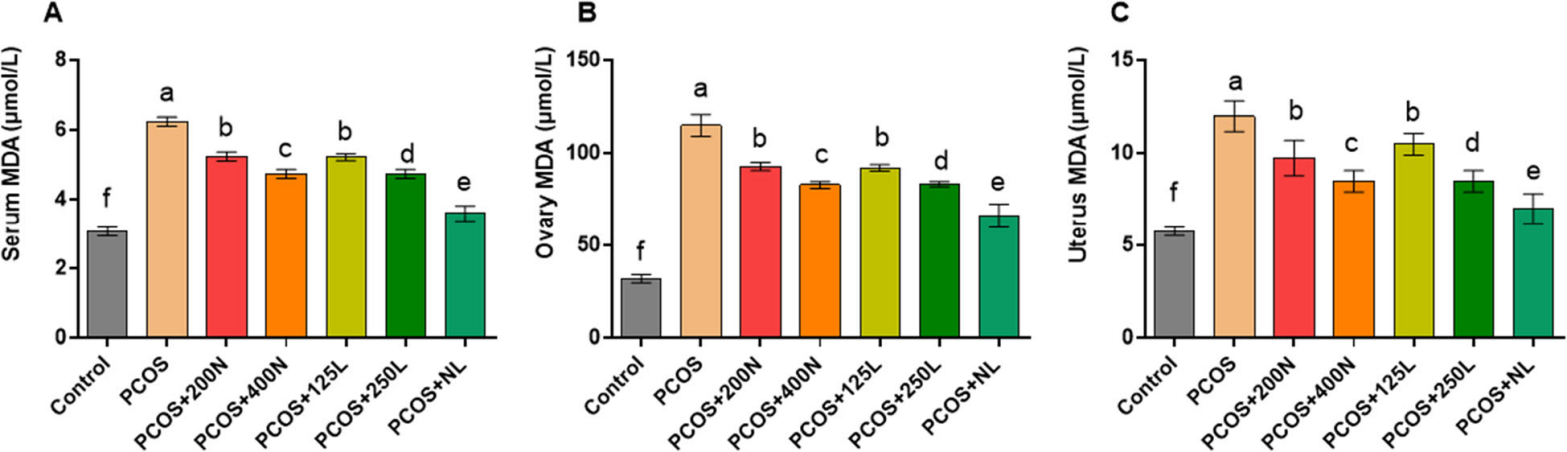
Malondialdehyde concentration in the serum, ovary, and uterine tissue samples are presented and compared in different groups of mice (*n* = 14)

Ovary MDA level: The lowest MDA levels were seen in the control group (32.00 ± 2.16 μmol/L) whereas the highest levels were recorded in the PCOS group (115.00 ± 5.77 μmol/L). Among the treatment groups, there was no significant difference between the PCOS+ N125 and PCOS+ N200 groups.

Uterus MDA level: The lowest MDA levels were in the control group (5.77 ± 0.22 μmol/L) and the highest levels were found in the PCOS group (125.00 ± 0.81 μmol/L). Among the treatment groups, there was no significant difference between the PCOS+ N125 and PCOS+ N200 groups.

### TAC levels in the serum, ovary, and uterus samples (Fig. 5)

Serum TAC concentration: The highest level of TAC was observed in the control group (0.265 ± 0.019 nmol/ml). Treatment with a combination of lutein and nettle could significantly increase the levels of TAC (0.220 ± 0.008 nmol/ml) in comparison with those of the PCOS group (*P* = 0.042). The lowest level of TAC was observed in the PCOS group (0.110 ± 0.008 nmol/ml). There were significant differences between all treatment groups (*P* < 0.05).

**Fig. 5:**
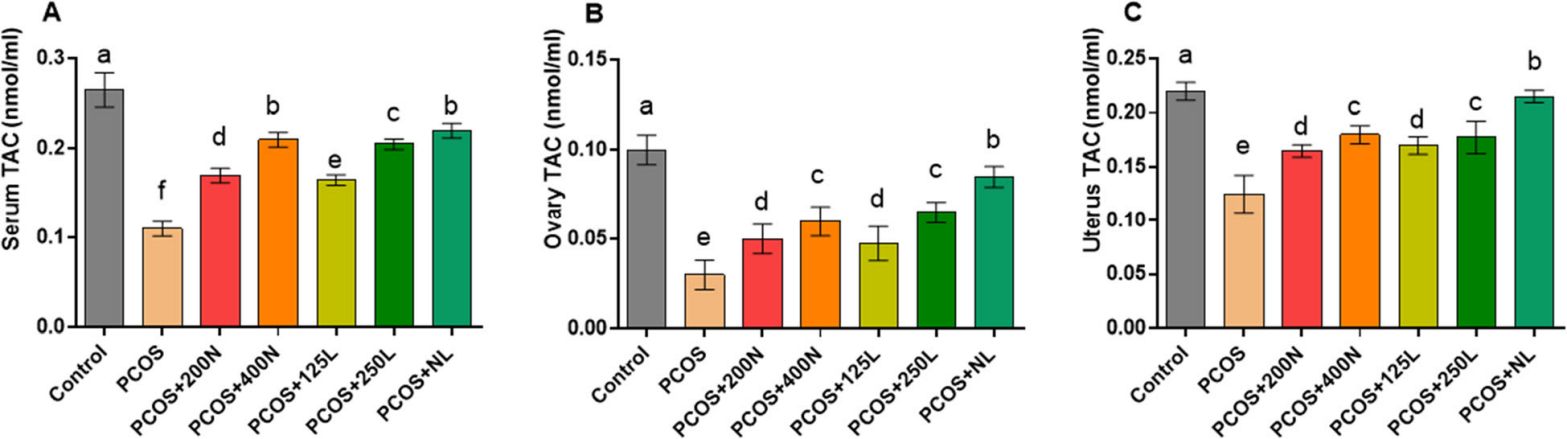
Total antioxidant capacity in the serum, ovary, and uterine tissue samples presented and compared in different groups of mice (*n* = 14)

Ovary TAC concentration: Among the treatment groups, there was no significant difference between the PCOS+ N125 and PCOS+ N200 groups. The results showed that in comparison with other treatments, a combination of lutein and nettle extract could result in the most favorable effect and significantly increased the TAC concentration (0.085 ± 0.005 nmol/ml) (*P* < 0.05).

Uterus TAC concentration: Here, all the results were similar to those observed for the ovary and serum levels of TAC. Despite the obvious difference between the PCOS+ N125 and PCOS+ N200 groups, the difference was not statistically significant.

### Superovulation and IVF

The results related to the superovulation, oviduct flushing, and in vitro fertilization rate, as well as the comparisons between different groups has presented in Table 1. The number of oocytes per animal in the PCOS group was significantly lower than that of the control group (*P* < 0.001). Among the treatment groups, the PCOS+ 400 N and PCOS+ 125 L groups had no significant difference with the control group. All treatment groups showed significant differences, except the PCOS+ 400 N and PCOS+ 125 L groups. There was a significant difference between the control group and all treatment groups, except for the PCOS+ 125 L group, in terms of the number of normal oocytes (*P* < 0.001). The highest number of abnormal oocytes was seen in the PCOS group which showed a significant difference with that of the treatment groups. The lowest number of abnormal oocytes among the treatment groups was observed in the PCOS+ 125 L and PCOS+NL groups. After the control group (92.79%), the highest percentage of fertilization was seen in the PCOS+ 125 L group (85.16%), but there was not a significant difference between the other treatment groups. Regarding the number of two-cell embryos, the PCOS+ 125 L group had the highest number of two-cell embryos, but there was no significant difference between the PCOS+ 125 L group and that of other treatment groups.

**Table 1:** Number of oocytes and embryos obtained following different treatments in the normal and PCOS groups

Group	N	Number of oocytes	Number of normal oocytes	Number of abnormal oocytes	Fertilization %	2-cell embryos	Blastocyst	Arrested embryo	Arrest type I	Arrest type II	Arrest type III
Control	8	227^a^	222 97.79%^a^	5 2.21%^e^	206 92.79%^a^	195 94.66%^a^	121 58.73%^a^	85 41.26%^c^	2 0.97%^d^	4 1.94%^d^	79 38.34%^c^
PCOS	8	91^e^	35 38.46%^c^	56 61.54%^a^	25 71.42%^c^	18 72%^c^	4 16%^d^	21 84%^a^	7 28%^a^	11 44%^a^	3 12%^d^
PCOS+ 200 N	8	214^b^	159 74.29%^b^	55 25.71%^b^	134 84.27%^b^	112 83.58%^b^	41 30.59%^b^	93 69.40%^b^	22 16.41%^b^	27 20.14%^b^	44 32.83%^b^
PCOS+ 400 N	8	238^a^	209 87.81%^ab^	29 12.19%^c^	178 85.16%^b^	151 84.83%^b^	56 31.46%^b^	122 68.53%^b^	15 8.42%^c^	28 15.73%^c^	79 44.38%^a^
PCOS+ 125 L	8	246^a^	222 90.24%^a^	24 9.76%^d^	197 88.73%^b^	173 87.81%^b^	55 27.91%^c^	142 72.08%^b^	19 9.64%^c^	34 17.25%^b^	89 45.17%^a^
PCOS+ 250 L	8	198^c^	169 85.35%^ab^	29 14.65%^c^	145 85.79%^b^	128 88.27%^b^	39 26.89%^c^	106 73.10%^b^	16 11.03%^c^	20 13.79%^c^	70 48.27%^a^
PCOS+NL	8	117^d^	93 79.48%^b^	24 20.52%^b^	80 86.02%^b^	69 86.25%^b^	25 31.25%^b^	55 68.75%^b^	9 11.25%^c^	17 21.25%^b^	29 36.25%^b^
*P*-values	–	***	***	***	***	***	***	***	***	***	***

Superscripts (***) show significant difference between the groups at *P* < 0.0001

^a, b, c, d^ Different superscript letters indicated significant differences in each column

A significant decrease in the blastocyst and two-cell rate was seen in the PCOS group in comparison with those of the control group (*P* = 0.0001). There was significant difference between the control and treatment groups. Among the treatment groups, the highest number of blastocysts was observed in the PCOS+ 125 L and PCOS+ 400 N groups. The percentage of embryos arrested at different stages of development increased significantly in the PCOS group compared with that of the control group. The quality of the majority of arrested embryos in the PCOS group was type II and III in compared to that of the controls (*P* = 0.0001). The administration of various concentrations of lutein and nettle extract improved the reproduction parameters, such as the number of oocytes, blastocyst, and embryo quality, and their morphology and reduced the percentage of arrested embryos compared to those of the PCOS group. A decrease was observed in the percentage of lysed and arrested embryos in the presence of all different doses of lutein and nettle extract compared with that of the PCOS group (*P* < 0.05).

## Discussion

In this study, PCOS induced oxidative stress in mice and decreased fertility by reducing the quality of the oocytes and embryos, but the oral administration of lutein and nettle extract, alone and in combination, improved reproductive function by increasing antioxidant activity, especially in the PCOS+ 125 L group. Both lutein and nettle extract increased TAC and decreased MDA levels in the PCOS mice.

PCOS as one of the causes of infertility, affects 4–8% of women during the reproductive age [48]. Ovarian follicles in PCOS have fewer granulosa cells per follicle in comparison to normal follicles [49]. Patients with PCOS show lower serum FSH levels compared to the normal cycles, and as a result of this deficiency, accumulation of antral follicles is seen in the ovary. High secretion of LH during the process of follicular development and differentiation can suppress the function of FSH, resulting in abnormal activity of granulosa cells [50]. In our results, estrus cycle was interrupted in metestrus phase in the DHEA-treated mice, it was suggested that their estrous cycles had been disrupted and serum luteinizing hormone levels were decreased. This process causes luteinizing structures before follicular maturation and atresia of small antral follicles in women with this syndrome. Short-term DHEA treatment (20 days) in contrast to long-term treatment may disrupt the central neuroendocrine regulatory mechanism and the ovaries were cystic with absent corpora lutea, showed hyperthecosis and luteinization of stroma, and had thickening of the tunica albugina [51].

Oxidative stress can cause embryonic damage. For example, ROS can diffuse through cell membranes and alter many types of cell molecules, including lipids, proteins, and nucleic acids. DHEA is an anabolic steroid that is converted to testosterone and dihydrotestosterone as an essential precursor in peripheral tissues [52]. Increased testosterone in this syndrome leads to increased oxidative stress, consequently, decreased zygote quality and caused mitochondrial damage, embryonic cell block, ATP deficiency and apoptosis by passing through the cell membrane. During in vitro culture of embryos, the free radicals production is more than the antioxidant capacity of embryos, then it can cause of growth arrest of embryos at different stages. Antioxidant capacity of embryos depends on their health, number and integrity of the blastomeres and membrane [14, 53]. In our study, TAC and MDA concentrations in blood, uterus and ovary were significantly lower and higher, respectively, in the PCOS group in comparison with those of the control. A high level of oxidative stress in patients with PCOS has harmful effects on oocyte maturation and embryo development. MDA has been reported as one of the lipid peroxidation products that rapidly mixes with biomolecules and disturbs glucose metabolism [54]. Oxidative stress is known to have an association with PCOS [11]. In studies on the relationship between oxidative stress and the incidence of PCOS, the researchers concluded that there was a direct correlation between malondialdehyde (MDA) and the incidence of PCOS [55].

Lutein and nettle extract showed antioxidant properties alone and in combination in serum, ovary, and uterus samples of normal and PCOS models of mice. Due to their high antioxidant properties and subsequent modulation of serum and tissue androgens, lutein and nettle extract reduce the destructive effects of the syndrome and thus improve fertility. Antioxidants could improve IVF rate and increase implantation [56]. In a study, lutein and zeaxanthin supplementation could decrease MDA concentration and increase TAC levels [39]. The antioxidant activity of the extract could be attributed to some compounds such as flavonoids, phenolic acids, and diterpenes [57]. Nettle extract contains antiandrogenic compounds, such as sterols, flavonoids, and polysaccharides [58]. Some studies have also determined the total antioxidant activity of the water extract of the nettle using the ferric thiocyanate method [31]. The combined form of nettle extract and lutein showed better antioxidant properties which could be attributed to their synergistic effects.

Estradiol (E2) modulates the function of reproductive organs and has positive and negative feedback on gonadotropin [59]. Phytoestrogen plays a role in antagonizing the E2 function [59]. It was reported that nettle extract could prevent the formation of dihydrotestosterone (the active form of testosterone) by inhibiting the enzyme 5-alpha reductase [60]. Nettle root extract could inhibit the aromatase activity, prevent androgen from binding to its receptors, and prevent the conversion of testosterone to estrogen [61]. The level of estradiol was significantly higher in the PCOS group in comparison to that of the control group, but the oral gavage of the nettle extract and lutein decreased estradiol concentration. The presence of many small follicles with a high estradiol concentration was first thought to cause a high rate of follicular atresia in the polycystic ovaries [62]. Increased estrogen could be attributed to the increased conversion of androgens to estrogen in the adipose tissues. It has been known that PCOS increases adipose tissue and that the increased adipose tissue can provide the substances which, in turn, can facilitate the increasing levels of estrogen [63]. Lutein and nettle extract, especially in a combined form, decreased the levels of estrogen. The authors could not find any study that had documented the effects of the lutein and nettle extract on estradiol concentration. Nettle extract can lead to an increase in the ovarian weight and the number of ovarian follicles (primary, secondary, and tertiary) which shows the positive effects of this extract in improving ovarian function [64]. Nettle extract caused blood vessels dilation and increased blood supply to tissues by increasing nitric oxide. Therefore, this extract can reduce atresia in primary and secondary follicles by increasing blood supply to ovaries and, then, increasing the perfusion of oxygen in the granulosa cells [65]. It was shown that nettle extract had positive effects on folliculogenesis by playing a direct role in increasing the estrogen hormonal levels [66]. Another study showed that the administration of *Urtica dioica* in women with hyperandrogenism lead to a decrease in the free and total serum testosterone levels; also, it could decrease significantly the DHEA level after treatment [67].

The adverse effects of PCOS on superovulation, fertilization, and embryo development have been reported in some studies. The patients with PCOS produce more oocytes during ovarian stimulation; these patients have poor oocyte quality and embryos and less cleavage and implantation rate and, thus, may suffer from higher abortion [10, 68, 69]. The results of present study showed that despite recovery of a high number of oocytes from PCOS mice after superovulation, the quality of oocytes were poor.

Both lutein and nettle extract improved the reproductive function in terms of oocyte recovery, oocyte maturation rate, fertilization, and percentage of blastocysts. Based on the findings of the present study, the best therapeutic response in terms of the number of oocytes, number of normal and abnormal oocytes, fertilization rate, number of two-cell embryos, and the number of blastocysts was observed in the PCOS+ 125 L group. Lutein has been associated with the antioxidant system in the reproduction system. An inappropriate antioxidant defense, as well as increased the production of ROS, can affect harmfully the reproductive function. The ROS not only is an important signal molecule that controls physiological activities including folliculogenesis, oocyte maturation, steroidogenesis, corpus luteal function, and luteolysis, in the female reproductive system [70], but also it has a significant role in the pathological processes in female reproduction [71, 72]. Carotenoids, along with other dietary antioxidants, could protect the body against extensive oxidative stress [73] and may help the reproductive system in an abnormal condition such as PCOS. The improved reproductive function in the group treated with nettle extract may also be attributed to its antioxidant activity.

Oral administration of lutein at a dose of 125 mg/kg and in a combination with nettle extract may be an option to decrease side effects of the PCOS syndrome. It is recommended to use more different doses and nettle extract from different seasons for future studies. The limitations of this study were lack of a treatment group as a reference medicine, HPLC analysis of nettle extract used in the study, measurement of testosterone concentration and calculation of estrogen:testosterone ratio, ultrastructural and ultrasound examination of ovaries after induction of PCOS in mice.

## Conclusions

PCOS induced oxidative stress in mice and decreased fertility by reducing the quality of the oocytes and embryos. Oral administration of lutein and nettle extract, alone and in combination, improved reproductive function by improving antioxidant activity. Treatment groups showed lower levels of MDA and estrogen but higher levels of TAC. Reproduction parameters such as the number of recovered oocyte, the number of normal oocyte, blastocyst and 2-cell embryos along with the fertilization rate were improved in treatment groups specially in combined and 125 mg/kg lutein groups.

## Data Availability

The datasets used and/or analyzed during the current study are available from the corresponding author on reasonable request.

## Abbreviations

PCOS: Polycystic ovary syndrome
DHEA: Dehydroepiandrosterone
MDA: Malondialdehyde
TAC: Total antioxidant capacity
ELISA: Enzyme linked immunoassay
ROS: Reactive oxygen species
IVF: In vitro fertilization
PMSG: Pregnant mare serum gonadotropin
hCG: Human chorionic gonadotropin
HTF: Human tubal fluid
BSA: Bovine serum albumin
ANOVA: Analysis of variance
SD: Standard deviation
TC: Total cholesterol
LDL-C: Low-density lipoprotein cholesterol
